# The Relationship Between Physical Activity and Mobile Phone Addiction in College Students: A Systematic Review and Meta-Analysis

**DOI:** 10.3390/bs15101325

**Published:** 2025-09-27

**Authors:** Laikang Yu, Zhuying Chen, Xiaorui Huang, Xifeng Tao, Yuanyuan Lv

**Affiliations:** 1 Beijing Key Laboratory of Sports Performance and Skill Assessment, Beijing Sport University, Beijing 100084, China; 2 Department of Strength and Conditioning Assessment and Monitoring, Beijing Sport University, Beijing 100084, China; zhuying20232120126@126.com (Z.C.); txf19983480529@126.com (X.T.); 3 Sports Coaching College, Beijing Sport University, Beijing 100084, China; 13115856760@163.com; 4 School of Physical Education, Xihua University, Chengdu 610039, China; 5 China Institute of Sport and Health Science, Beijing Sport University, Beijing 100084, China

**Keywords:** physical activity, mobile phone addiction, depression, anxiety, self-control, sleep quality, academic burnout, procrastination, college students

## Abstract

This study aimed to elucidate the relationship between physical activity (PA) and mobile phone addiction (MPA) in college students. Five databases (PubMed, Scopus, Embase, Web of Science, and Cochrane) were searched up to 20 January 2025. A random-effects meta-analysis was conducted to calculate combined Pearson correlation coefficients (r) with 95% confidence intervals. A total of 29 studies were included in the analysis. A significant negative correlation was found between PA and MPA (r = −0.349; *p* < 0.001). Subgroup analyses revealed a larger effect size in alleviating MPA after the COVID-19 pandemic (r = −0.340; *p* = 0.008). Additionally, PA demonstrated a large effect size in improving sleep quality (r = −0.365; *p* < 0.001) and reducing depression and anxiety (r = −0.356; *p* = 0.024). The effect of PA on self-control was moderate (r = −0.267; *p* < 0.001), as was its effect on procrastination (r = −0.330; *p* = 0.016). In contrast, the effect of PA on academic burnout was small (r = −0.141; *p* < 0.001). In conclusion, increasing PA may reduce MPA by alleviating depression and anxiety and enhancing self-control. PA’s benefits for MPA extend to improving sleep quality and reducing academic burnout and procrastination.

## 1. Introduction

College students constitute a distinct demographic undergoing the pivotal transition from adolescence to adulthood (Pedrelli et al., 2015), a developmental stage termed emerging adulthood (ages 18 to 25) (Arnett, 2000). This phase is marked by frequent life changes and the exploration of identity possibilities, and it is characterized by comparatively lower maturity levels than older populations (Parker et al., 2004). During this semi-autonomous period, which is often distinguished by underdeveloped self-regulation, college students are particularly susceptible to mental health challenges (Arnett et al., 2014). Notably, mobile phone addiction (MPA) has emerged as a salient issue in digital-native students (Palfrey & Gasser, 2008). MPA is defined as a compulsive behavioral pattern involving uncontrolled mobile phone use that compromises physical, psychological, and social functioning (Chóliz, 2010). Global prevalence of MPA is alarming: 34.6% in Turkey (Dikeç & Kebapçı, 2018), 44.7% in South India (Kumar et al., 2019), 64.6% in America (Lepp et al., 2013), 33.6% in China (Mei et al., 2023), and 56.7% in Jordan (Abuhamdah & Naser, 2023). From 2019 to 2024, global mobile phone ownership increased by 114.97%, and it is projected to reach 4.88 billion users by 2025 (BankMyCell, 2025). Substantial evidence links MPA to academic underperformance (Kates et al., 2018) and systemic deterioration across multiple health domains, including physical well-being (Berolo et al., 2011; Lepp et al., 2013), psychological stability (Abuhamdah & Naser, 2023; Winkler et al., 2020), and sleep quality (Y. Li et al., 2020; Mei et al., 2023; Morita & Sasai-Sakuma, 2023), positioning it as a worldwide public health crisis.

Current interventions emphasize cost-effective strategies such as physical activity (PA) to mitigate MPA. As a non-pharmacological intervention, PA demonstrates therapeutic potential in reducing mobile phone dependence (S. J. Liu et al., 2019) while addressing MPA-related comorbidities such as depression and anxiety (Ji et al., 2024), sleep disturbances (Gao et al., 2023), and sedentary behaviors (Lai et al., 2025; Pirwani & Szabo, 2024). Previous studies have shown that in adolescent and young adult populations, the intensity of mobile phone use is negatively correlated with PA (Lai et al., 2025; Xiao et al., 2022). Specifically, Lai et al. (2025) identified moderate-to-vigorous PA as effective in alleviating MPA in college students, whereas a six-month longitudinal study found no significant PA-MPA correlation (Huang et al., 2022). A recent meta-analysis further reported only weak direct associations between PA and MPA in college students (Pirwani & Szabo, 2024), highlighting the need for a clearer understanding of the underlying mechanisms.

Despite the public health urgency of MPA, meta-analytic investigations remain limited. Five meta-analyses conducted since 2019 have revealed inconsistent conclusions: one found mobile phones effective for PA promotion in adults (Feter et al., 2019), another observed reduced PA levels among excessive phone uses (particularly ages 13–25) (Zagalaz-Sánchez et al., 2019), two studies examined the effects of exercise interventions on MPA (Z. X. Li et al., 2023; S. J. Liu et al., 2019), and the last study analyzed the relationship between PA and MPA in adolescents and young adults (Xiao et al., 2022). These studies are impacted by methodological limitations, including small sample sizes, high heterogeneity, and insufficient analysis of moderating variables.

Notably, only one recent systematic review concluded that PA is effective in reducing MPA in college students, but it omitted an exploration of the mechanisms involved. Therefore, this study aimed to elucidate the complex relationship between PA and MPA in college students and to address existing research gaps through a comprehensive analysis. Specifically, this study sought to answer the following research questions: (1) Does PA significantly reduce MPA in college students? (2) Through what psychological or physiological mechanisms does PA exert its effects on MPA?

## 2. Materials and Methods

This study adhered to the Preferred Reporting Items for Systematic Reviews and Meta-Analyses guidelines (PRISMA) (Page et al., 2021) and was registered with the International Prospective Register of Systematic Reviews (PROSPERO, CRD420250652238).

### 2.1. Search Strategy

Searches of the databases PubMed, Web of Science, Scopus, Embase, and Cochrane Library were conducted. The search encompassed studies published up to 20 January 2025, using the keywords and Medical Subject Headings (MESH) terms: “physical activity” and “mobile phone addiction” (Table S1). Two authors (L.Y. and Z.C.) independently screened titles, abstracts, and full texts for eligibility. Discrepancies were resolved through discussion with a third author (Y.L.).

### 2.2. Inclusion and Exclusion Criteria

The Population, Intervention, Comparison, Outcome (PICO) framework was utilized to establish the inclusion criteria as follows: (a) Population: college students; (b) Intervention: quantitative observational studies that assessed PA levels in college students; (c) Comparison: studies that evaluated the relationships between PA and multiple variables, including MPA, sleep quality, self-control, depression and anxiety, academic burnout, and procrastination; (d) Outcome: the primary outcome was MPA, whereas the secondary outcomes included sleep quality, self-control, depression and anxiety, academic burnout, procrastination.

Studies were excluded if they: (1) were reviews, case reports, or non-peer-reviewed works; (2) included participants with diagnosed psychiatric, orthopedic, or neurologic conditions that impaired PA participation; and (3) lacked extractable correlation values.

### 2.3. Data Extraction

Two authors independently extracted data using standardized forms, capturing the following information: (1) study details (title, author, year); (2) sample characteristics (size, gender distribution, age, academic grade, major); (3) data collection timeline; (4) PA and MPA measurement methods; and (5) the primary outcome (MPA) and secondary outcomes (sleep quality, self-control, depression and anxiety, academic burnout, procrastination).

### 2.4. Quality Assessment

Two authors evaluated the quality of the selected cross-sectional studies using the criteria from the Joanna Briggs Institute (JBI) appraisal checklist (Moola et al., 2015), which comprises 10 items scored from 0 to 2. Studies with an overall score exceeding 70% were deemed high quality and low risk of bias, while those below this threshold were considered low quality and high risk of bias. Notably, quality assessment scores did not affect study inclusion. Publication bias was assessed via funnel plot, with *p* < 0.05 indicating significant publication bias.

### 2.5. Statistical Analysis

All statistical analyses were conducted using Comprehensive Meta-Analysis software (version 3; Biostat Inc.; Englewood, NJ, USA). The correlation coefficient (r) was used to assess the relationship between PA and MPA. Combined Pearson correlation coefficients (with 95% confidence intervals [CIs]) were calculated using the inverse variance method and converted to Fisher z-scores to stabilize variance. Effect sizes were interpreted as small (r = 0.1–0.2), medium (r = 0.2–0.35), and large (r > 0.35) (Polanin & Snilstveit, 2016). Due to significant heterogeneity across studies, a random-effects model was employed. Heterogeneity was quantified using I^2^ index and Cochran Q statistic, categorized as low (I^2^ ≤ 25%), medium (25% < I^2^ ≤ 50%), high (50% < I^2^ ≤ 75%), and very high (I^2^ > 75%). Within-subgroup significance was assessed via Z-tests, while between-subgroup differences were evaluated using Q-tests (Higgins & Thompson, 2002). Sensitivity analyses were performed by iteratively excluding one study at a time to assess the stability of the correlation coefficients between PA and MPA.

## 3. Results

### 3.1. Study Selection

**Figure 1:**
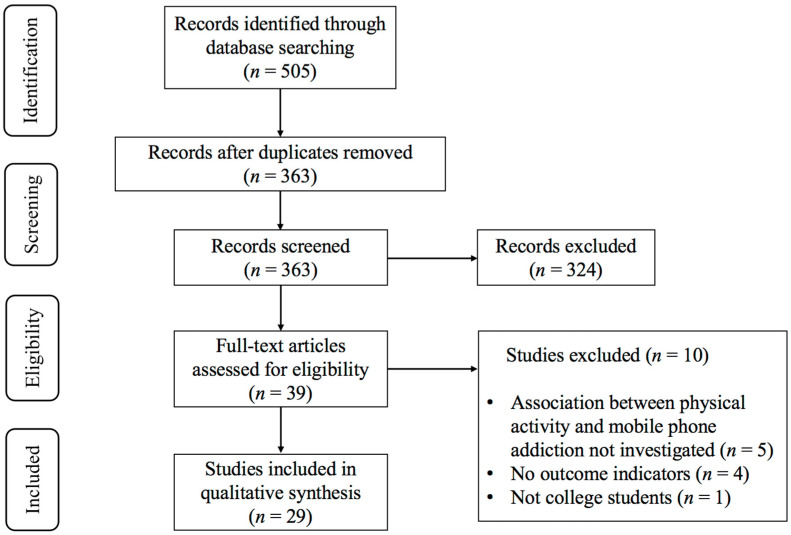
PRISMA flowchart of study selection.

As depicted in Figure 1, the initial search identified 505 records from PubMed (*n* = 50), Scopus (*n* = 323), Embase (*n* = 39), Web of Science (*n* = 83), and Cochrane Library (*n* = 10). After removing duplicates, 363 records remained. Following title and abstract screening, 39 potentially eligible studies were identified. Ten studies were excluded for the following reasons: (1) non-college student populations (*n* = 1), (2) absence of PA-MPA relationship reporting (*n* = 5), (3) lack of outcome indicators (*n* = 4). Finally, 29 studies (Buke et al., 2021; Chen et al., 2022; Gong et al., 2023; Guo et al., 2022; Haripriya et al., 2019; Han et al., 2023; Huang et al., 2022; Jin et al., 2023; Ke et al., 2023; Kim et al., 2015; C. Liu & Sun, 2023; Meng et al., 2024, 2025; Numanoğlu-Akbaş et al., 2020; Saffari et al., 2022; Song et al., 2024; Tao et al., 2024; W. X. Tong et al., 2022; W. Tong & Meng, 2023; J. Wang et al., 2024; Q. Wang et al., 2024; J. Wu et al., 2024; Yang et al., 2021; Yin et al., 2024; Zeng et al., 2022; Zhao et al., 2022; Zhao & Kou, 2023; L. Zhu et al., 2023; W. Zhu et al., 2024) were included in this systematic review and meta-analysis.

### 3.2. Characteristics of the Included Studies

The characteristics of the 29 studies are summarized in Table S2. The total sample size was 54,039. Among these, 28 studies (Buke et al., 2021; Chen et al., 2022; Gong et al., 2023; Guo et al., 2022; Haripriya et al., 2019; Han et al., 2023; Huang et al., 2022; Jin et al., 2023; Ke et al., 2023; Kim et al., 2015; C. Liu & Sun, 2023; Meng et al., 2024, 2025; Numanoğlu-Akbaş et al., 2020; Song et al., 2024; Tao et al., 2024; W. X. Tong et al., 2022; W. Tong & Meng, 2023; J. Wang et al., 2024; Q. Wang et al., 2024; J. Wu et al., 2024; Yang et al., 2021; Yin et al., 2024; Zeng et al., 2022; Zhao et al., 2022; Zhao & Kou, 2023; L. Zhu et al., 2023; W. Zhu et al., 2024) included both male and female participants, while one study (Saffari et al., 2022) included only females. Twenty-five studies (Chen et al., 2022; Gong et al., 2023; Guo et al., 2022; Haripriya et al., 2019; Han et al., 2023; Huang et al., 2022; Jin et al., 2023; Ke et al., 2023; C. Liu & Sun, 2023; Meng et al., 2024, 2025; Saffari et al., 2022; Song et al., 2024; Tao et al., 2024; W. X. Tong et al., 2022; W. Tong & Meng, 2023; J. Wang et al., 2024; Q. Wang et al., 2024; J. Wu et al., 2024; Yang et al., 2021; Yin et al., 2024; Zeng et al., 2022; Zhao et al., 2022; Zhao & Kou, 2023; L. Zhu et al., 2023; W. Zhu et al., 2024) were conducted in China, two (Numanoğlu-Akbaş et al., 2020; Buke et al., 2021) in Turkey, and one (Kim et al., 2015) in South Korea. Three studies (Haripriya et al., 2019; Kim et al., 2015; Numanoğlu-Akbaş et al., 2020) collected data before coronavirus disease 2019 (COVID-19), 19 studies (Buke et al., 2021; Chen et al., 2022; Gong et al., 2023; Guo et al., 2022; Han et al., 2023; Huang et al., 2022; Ke et al., 2023; C. Liu & Sun, 2023; Saffari et al., 2022; W. X. Tong et al., 2022; W. Tong & Meng, 2023; J. Wang et al., 2024; J. Wu et al., 2024; Yang et al., 2021; Zeng et al., 2022; Zhao et al., 2022; Zhao & Kou, 2023; L. Zhu et al., 2023; W. Zhu et al., 2024) during COVID-19, and 7 studies (Jin et al., 2023; Meng et al., 2024, 2025; Song et al., 2024; Tao et al., 2024; Q. Wang et al., 2024; Yin et al., 2024) after COVID-19.

### 3.3. Effect of PA on MPA in College Students

**Figure 2:**
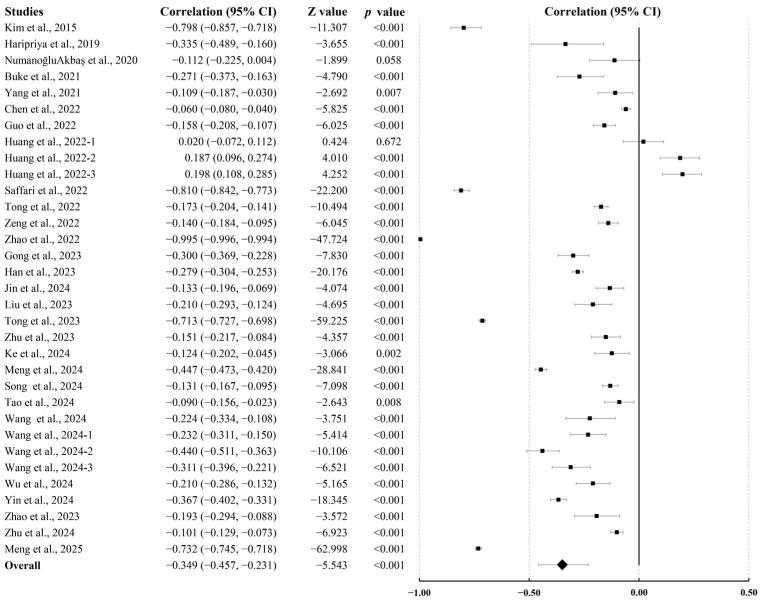
Summary of pooled correlation between PA and MPA in college students (Buke et al., 2021; Chen et al., 2022; Gong et al., 2023; Guo et al., 2022; Haripriya et al., 2019; Han et al., 2023; Huang et al., 2022; Jin et al., 2023; Ke et al., 2023; Kim et al., 2015; C. Liu & Sun, 2023; Meng et al., 2024, 2025; Numanoğlu-Akbaş et al., 2020; Saffari et al., 2022; Song et al., 2024; Tao et al., 2024; W. X. Tong et al., 2022; W. Tong & Meng, 2023; J. Wang et al., 2024; Q. Wang et al., 2024; J. Wu et al., 2024; Yang et al., 2021; Yin et al., 2024; Zeng et al., 2022; Zhao et al., 2022; Zhao & Kou, 2023; L. Zhu et al., 2023; W. Zhu et al., 2024).

Thirty-three independent data points were included in the meta-analysis. Due to high heterogeneity (Q = 7017.177, *p* < 0.001; I^2^ = 99.544), a random-effects model was applied. The pooled correlation coefficient was r = −0.349 (95% CI, −0.457 to −0.231; *p* < 0.001; Figure 2), indicating a moderate negative association between PA and MPA.

**Figure 3:**
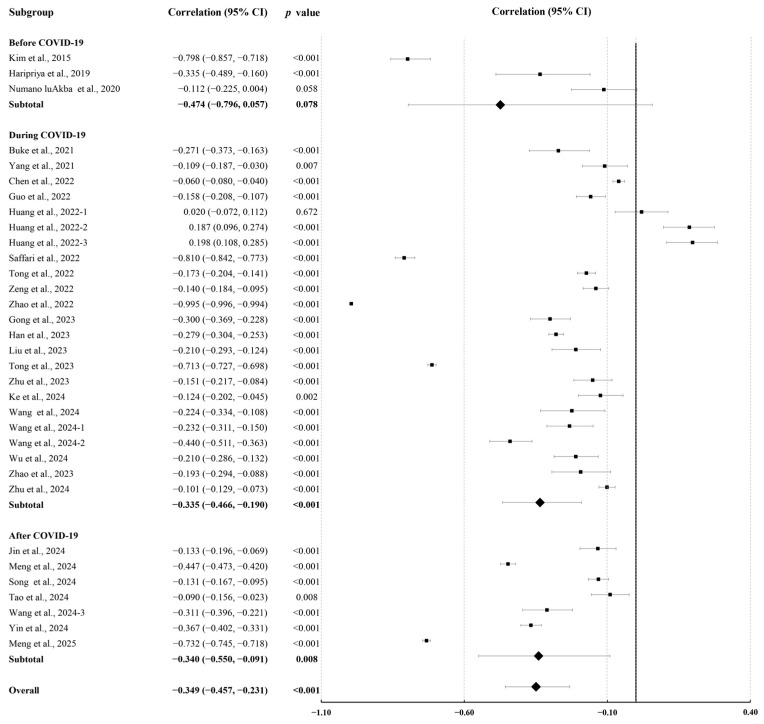
Subgroup analyses of summary correlation between PA and MPA in college students before, during, and after COVID-19 (Buke et al., 2021; Chen et al., 2022; Gong et al., 2023; Guo et al., 2022; Haripriya et al., 2019; Han et al., 2023; Huang et al., 2022; Jin et al., 2023; Ke et al., 2023; Kim et al., 2015; C. Liu & Sun, 2023; Meng et al., 2024, 2025; Numanoğlu-Akbaş et al., 2020; Saffari et al., 2022; Song et al., 2024; Tao et al., 2024; W. X. Tong et al., 2022; W. Tong & Meng, 2023; J. Wang et al., 2024; Q. Wang et al., 2024; J. Wu et al., 2024; Yang et al., 2021; Yin et al., 2024; Zeng et al., 2022; Zhao et al., 2022; Zhao & Kou, 2023; L. Zhu et al., 2023; W. Zhu et al., 2024).

Post-COVID-19 data showed slightly stronger correlations (r = −0.340; 95% CI, −0.550 to −0.091; *p* = 0.008) compared to during-COVID-19 data (r = −0.335; 95% CI, −0.466 to −0.190; *p* < 0.001; Figure 3).

### 3.4. Effect of PA on Sleep Quality in College Students

**Figure 4:**
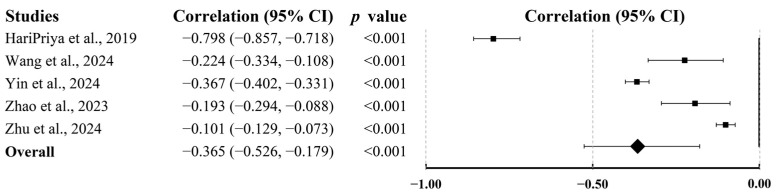
Summary of pooled correlation between PA and sleep quality in college students (Haripriya et al., 2019; J. Wang et al., 2024; Yin et al., 2024; Zhao & Kou, 2023; W. Zhu et al., 2024).

Five studies involving 7665 participants evaluated sleep quality. Pooled analysis demonstrated a significant negative association between PA and sleep quality (r = −0.365; 95% CI, −0.526 to −0.179; *p* < 0.001; Figure 4), indicating a large effect size.

### 3.5. Effect of PA on Self-Control in College Students

**Figure 5:**
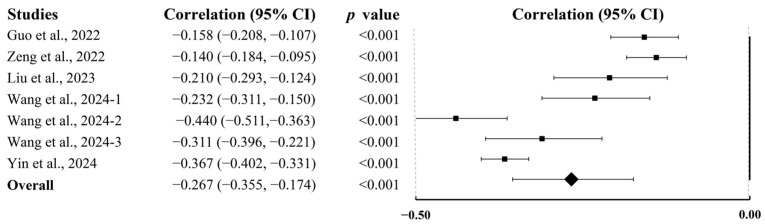
Summary of pooled correlation between PA and self-control in college students (Guo et al., 2022; C. Liu & Sun, 2023; Q. Wang et al., 2024; Yin et al., 2024; Zeng et al., 2022).

Seven studies involving 7441 participants evaluated self-control. Pooled analysis revealed a medium negative association between PA and self-control (r = −0.267; 95% CI, −0.355 to −0.174; *p* < 0.001; Figure 5).

### 3.6. Effect of PA on Depression and Anxiety in College Students

**Figure 6:**
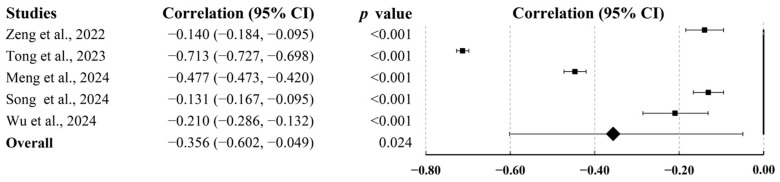
Summary of pooled correlation between PA and depression and anxiety in college students (Meng et al., 2024; Song et al., 2024; W. X. Tong et al., 2022; J. Wu et al., 2024; Zeng et al., 2022).

Five studies involving 13,336 participants evaluated depression and anxiety. Pooled analysis identified a large negative association between PA and depression and anxiety (r = −0.356; 95% CI, −0.602 to −0.049; *p* = 0.024; Figure 6).

### 3.7. Effect of PA on Academic Burnout in College Students

**Figure 7:**
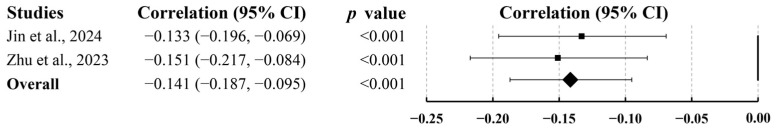
Summary of pooled correlation between PA and academic burnout in college students (Jin et al., 2023; L. Zhu et al., 2023).

Two studies involving 1753 participants evaluated academic burnout. Pooled analysis showed a small negative association between PA and academic burnout (r = −0.141; 95% CI, −0.187 to −0.095; *p* < 0.001; Figure 7).

### 3.8. Effect of PA on Procrastination in College Students

**Figure 8:**
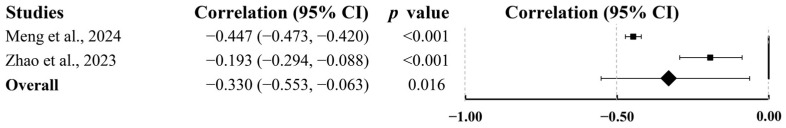
Summary of pooled correlation between PA and procrastination in college students (Meng et al., 2024; Zhao & Kou, 2023).

Two studies involving 3936 participants evaluated procrastination. Pooled analysis reported a medium negative association between PA and procrastination (r = −0.330; 95% CI, −0.533 to −0.063; *p* = 0.016; Figure 8).

### 3.9. Risk of Bias

The methodological quality assessment results are presented in Table S3. The mean JBI score was 16.55 (85.6%), indicating high-quality studies with low risk of bias.

### 3.10. Publication Bias

The funnel plot illustrated a symmetrical distribution of effect sizes against their standard errors. Consistently, Egger’s test indicated no evidence of significant publication bias (*p* = 0.41, Figure S1), suggesting that the results of this meta-analysis are unlikely to be influenced by selective publication.

### 3.11. Sensitivity Analysis

When each study was removed one at a time, the correlation coefficients ranged from r = −0.275 to −0.364 (Figure S2). This demonstrated that no single study unduly influenced the overall meta-analysis results, confirming the reliability of our findings.

## 4. Discussion

To our knowledge, this study represents the first systematic review and meta-analysis to investigate the relationship between PA and MPA in college students. Our findings revealed a significant and negative correlation between PA and MPA (r = −0.349). This result is consistent with several studies that have reported a clear negative relationship between PA and MPA (Kim et al., 2015; Meng et al., 2025; Q. Wang et al., 2024). For instance, Yang et al. (2021) identified PA as a protective factor against MPA in college students, and another cross-sectional study concluded that PA protects adolescents from MPA (W. Wu et al., 2024).

From the perspective of the ternary interaction theory, PA, as an external environmental stimulus, plays a crucial role in reducing behavioral addictions (Bandura, 1989). Empirical evidence indicates that PA significantly reduces MPA in young people (Azam et al., 2020). Mechanistically, PA can divert attention from negative emotions, thereby reducing reliance on mobile phones for emotional regulation. Neurophysiologically, PA mitigates addiction behaviors by enhancing neuroplasticity and cognitive functioning (Scholler et al., 2023), reducing impulsivity, improving emotion regulation, and decreasing cravings (Cabé et al., 2021). Specifically, long-term PA increases neuroplasticity in brain regions associated with reward processing and diminishes MPA through effects on reward strategies (Cassilhas et al., 2016). However, some studies have reported no significant association between PA and MPA. For instance, one study found no link between PA and mobile phone use in college students from different majors (Barkley & Lepp, 2016), and a six-month longitudinal study also found no significant correlation between PA and MPA (Huang et al., 2022). Furthermore, a recent meta-analysis revealed only a weak direct relationship between PA and MPA in college students (Pirwani & Szabo, 2024). These inconsistencies may stem from variations in sample sizes, geographical regions, and measurement methods. For instance, Xiao et al. (2022) found a moderate negative correlation between PA and MPA in young people, unaffected by data collection timing, country, or population type. Another study comparing PA and mobile phone use in American and Thai college students showed a significant negative relationship in Thai college students but not in American students (Penglee et al., 2019). Thus, further exploration of the mechanisms underlying PA’s impact on MPA is warranted.

The time of data collection, particularly the impact of the COVID-19 pandemic, is a critical factor in the relationship between PA and MPA. Our subgroup analysis indicated no significant correlation between PA and MPA before COVID-19 but a significant correlation during and after the pandemic, with a stronger correlation post-pandemic. The COVID-19 pandemic and related restrictions have disrupted the health and lifestyle behaviors of college students globally, leading to decreased PA and increased sedentary and mobile phone use (Gallè et al., 2020; López-Valenciano et al., 2021; Motevalli et al., 2023; Savage et al., 2024; B. Zhang et al., 2022). According to the theory of compensatory internet use, individuals in negative life situations are more likely to use the internet or mobile phones to alleviate negative emotions (Kardefelt-Winther, 2014). This has resulted in increased levels of depression, anxiety, and stress in college students while decreasing PA (Huckins et al., 2020; Wolf et al., 2021; Y. Zhang et al., 2023). Consequently, students are more likely to rely on mobile phones to cope with these negative emotions (Huckins et al., 2020). Thus, the role of PA in reducing MPA became more pronounced during the pandemic. Post-pandemic, as socialization and PA resumed, PA levels increased (Yun & Lee, 2023), potentially explaining its enhanced effectiveness of PA in reducing MPA. This suggests that pandemic-induced lifestyle changes and mental health issues significantly impacted both PA and MPA, thereby influencing the relationship.

Our results also revealed a large effect size for depression and anxiety in the relationship between PA and MPA (r = −0.356), indicating that PA can reduce MPA by alleviating depressive and anxious symptoms. Previous studies have demonstrated that negative emotions such as loneliness (Zeng et al., 2022), interpersonal distress (C. Liu & Sun, 2023), low self-esteem (Ke et al., 2023), stress (Zhao et al., 2022), and depression and anxiety (W. Tong & Meng, 2023) can influence the relationship between PA and MPA. Our findings support the mediating role of depression and anxiety in this relationship, aligning with previous studies. Some studies have confirmed that psychological distress mediates the relationship between PA and MPA (Song et al., 2024; Zeng et al., 2022), while others have identified pathways where negative emotions serve as mediators or chain mediators between PA and MPA (W. Tong & Meng, 2023; J. Wu et al., 2024). Common to these studies is the positive effect of PA interventions on depression and anxiety (Mundell et al., 2024). A possible physiological explanation involves PA’s ability to reverse stress-induced corticosterone abnormalities in the adrenal glands, hippocampus, and plasma, helping to maintain normal hypothalamic–pituitary–adrenal (HPA) axis function (Heijnen et al., 2015; S. Li et al., 2020; Stranahan et al., 2008). Additionally, PA upregulates monoamine neurotransmitters like dopamine and norepinephrine and promotes striatal plasticity (Dimsdale & Moss, 1980; S. J. Liu et al., 2019). Therefore, PA reduces stress and anxiety by increasing norepinephrine and endorphin levels, thereby decreasing MPA.

Self-control emerged as a significant mediator in the relationship between PA and MPA (r = −0.0267). This finding is consistent with other studies. Previous studies have shown that self-control fully mediates the relationship between physical education classes and MPA in college students (Guo et al., 2022; Zeng et al., 2022; Zhong et al., 2021). Other studies have demonstrated that PA can enhance self-control and reduce the chain-mediated effects of MPA in college students (C. Liu & Sun, 2023; J. Wang et al., 2024; Yin et al., 2024). Self-control, defined as the ability to regulate responses and overcome impulses, is a key psychological predictor of MPA (Bianchi & Phillips, 2005; Tangney et al., 2004). Neurobiological studies have indicated that PA can control reward impulses and alleviate MPA by altering dopamine circuits in the mesolimbic and nigrostriatal pathways (Greenwood, 2019; Herrera et al., 2016). PA also improves cognitive motivation by enhancing central nervous system (CNS) structure and connectivity, increasing hippocampal volume, and promoting prefrontal cortex growth, thereby reducing MPA (Soga et al., 2025; Weinstein et al., 2012; Voss et al., 2010). Furthermore, PA’s reduction in MPA through self-control can be explained by the self-control resource model (Baumeister et al., 1998). Self-control is a limited resource that can be depleted after use (Baumeister & Vohs, 2016). When PA and MPA are viewed as long-term stable behaviors, training and endurance enhancement can increase self-control resilience beyond its original limitations. This improves an individual’s self-control traits and reduces MPA (Zhong et al., 2021). Self-control can effectively inhibit unhealthy phone use, while PA can enhance college students’ control (Kinnunen et al., 2012). Thus, self-control plays a crucial mediating role in PA’s effects on MPA.

As expected, PA’s positive impact on MPA also extends to improving sleep quality (r = −0.365), reducing academic burnout (r = −0.141), and decreasing procrastination (r = −0.141), with the most significant improvements observed in sleep quality. This is consistent with previous studies. J. Wang et al. (2024) suggested that PA improves sleep quality directly and by reducing stress, which further alleviates MPA. Additionally, Yin et al. (2024) explored how PA affects sleep quality by enhancing self-control and reducing MPA. Furthermore, Zhao and Kou (2023) confirmed PA’s role in reducing MPA and procrastination behaviors and improving sleep quality. When college students are immersed in mobile phone use, their nervous system becomes overactive, producing excitatory hormones that disrupt circadian rhythms and affect sleep quality (Parekh & McClung, 2015). Since young people with MPA often report more sleep deprivation (Thomée et al., 2011) and academic burnout (Jin et al., 2023), PA may improve sleep quality and procrastination by stimulating the pituitary gland to release more endorphins. These endorphins can compete with addictive substances for receptors in the CNS, thereby inhibiting addiction (Everly & Lating, 2002). Consequently, PA’s improvement of MPA effectively enhances sleep quality, reduces academic burnout, and decreases procrastination in college students.

Numerous other factors may influence the relationship between PA and MPA. The role of gender remains inconclusive. Some studies indicated that gender moderates the relationship between social anxiety and MPA in medical students, with males more likely than females to use mobile phones to relieve anxiety (Song et al., 2024). This may be because females tend to seek social support to alleviate anxiety when experiencing social distress (Chukwuere & Chukwuere, 2017; Song et al., 2024). However, other research suggested that female participants may be more dependent on mobile phones than males (Xu et al., 2022). Reasons for this include higher weight-related self-stigma, greater use of social media for emotional expression, and lower PA levels in females (C. Liu & Sun, 2023). Specifically, male college students generally engage in PA at higher levels, intensity, duration, and frequency than female students (W. X. Tong et al., 2022). Additionally, significant differences exist in the relationship between college students’ majors and grades (W. Tong & Meng, 2023). Thus, future research should continue to explore individual difference factors.

Despite being the first meta-analysis to assess the relationship between PA and MPA in college students, some limitations persist. First, this study included a limited number of studies (*n* = 29), all of which were cross-sectional. As more original research becomes available, it may be necessary to re-examine the evidence for the association between college students’ PA and MPA and the moderating factors involved. Second, although all studies included in this review were conducted in Asia, this provides a valuable perspective on the cultural and contextual factors shaping the relationship between PA and MPA in this region. However, the findings may not be fully generalizable to other cultural contexts. Future research should therefore compare evidence from Asia with studies conducted in Western and other regions to examine potential cross-cultural differences. Additionally, data limitations from the included studies may subjectively influence the estimated effect sizes. Finally, socio-cultural differences in the samples and variations in measurement methods may contribute to discrepancies between studies. Despite efforts to control for potential biases, these limitations could not be entirely avoided.

## 5. Conclusions

PA exhibited a moderate negative correlation with MPA in college students, with stronger effects observed in the post-pandemic context. Increasing PA may reduce MPA by alleviating depression and anxiety, enhancing self-control, improving sleep quality, and mitigating academic burnout and procrastination. Academically, these findings contribute to a deeper understanding of the psychological mechanisms linking PA and MPA, highlighting PA as a multidimensional protective factor. Practically, the results suggest that universities and policymakers should integrate structured PA programs into student health promotion strategies as a feasible, non-pharmacological approach to reducing MPA and supporting mental well-being.

## Data Availability

The original contributions presented in the study are included in the article/Supplementary Materials, further inquiries can be directed to the corresponding author.

## Supplementary Materials

The following supporting information can be downloaded at: https://www.mdpi.com/article/10.3390/bs15101325/s1, Figure S1: Funnel plot of MPA; Figure S2: Sensitivity analysis results of MPA; Table S1: Search strategies; Table S2: Characteristics of the studies included in this meta-analysis; Table S3: Details of the scoring criteria in the JBI appraisal checklist.

## Abbreviations

The following abbreviations are used in this manuscript:

CI	Confidence interval
CNS	Central nervous system
HPA	Hypothalamic–pituitary–adrenal
JBI	Joanna Briggs Institute
MESH	Medical Subject Headings
MPA	Mobile phone addiction
PA	Physical activity
PICO	Population, Intervention, Comparison, Outcome
PRISMA	Preferred Reporting Items for Systematic Reviews and Meta-Analyses
