# Supplementary material for: The Relationship Between Physical Activity and Mobile Phone Addiction in College Students: A Systematic Review and Meta-Analysis
Source: Behav Sci (Basel). 2025 Sep 27;15(10):1325. doi: 10.3390/bs15101325 (PMC12561036; doi:10.3390/bs15101325)

Supplemental material

The relationship between physical activity and mobile phone addiction in college students: a systematic review and meta-analysis

Table S1. Search strategies 2

Table S2. Characteristics of the studies included in this meta-analysis 3

Table S3. Details of the scoring criteria in the JBI appraisal checklist 7

Figure S1. Funnel plot of MPA...10

Figure S2. Sensitivity analysis results of MPA 11

Table S1. Search strategies

**Physical activity search terms combined with "OR"**
Physical Activity; Activities, Physical; Activity, Physical; Physical Activities; Exercise; Exercises; Exercise, Physical; Exercises, Physical; Physical Exercise; Physical Exercises; Exercise, Aerobic; Aerobic Exercise; Aerobic Exercises; Exercises, Aerobic; Exercise, Isometric; Exercises, Isometric; Isometric Exercises; Isometric Exercise; Acute Exercise; Acute Exercises; Exercise, Acute; Exercises, Acute; Exercise Training; Exercise Trainings; Training, Exercise;Trainings, Exercise
**Mobile phone addiction search terms combined with "OR"**
Mobile Phone Addiction; Technology Addiction; Technology Addictions; Addiction, Mobile Phone; Addictions, Mobile Phone; Mobile Phone Addictions; Phone Addiction, Mobile; Cell Phone Addiction; Addiction, Cell Phone; Cell Phone Addictions; Video Game Addiction; Addictions, Video Game; Addiction, Video Game; Game Addiction, Video; Video Game Addictions

Table S2. Characteristics of the studies included in this meta-analysis.

Study	Country	Sample size (male/female)	Age (year)	Time	Measurement		*r*
					MPA	PA	
Kim et al., 2015	South Korea	110 (67/43)	21.03 ± 1.61	2015	SAPS	Sensor Pedometer	-0.798
Haripriya et al., 2019	India	113 (63/50)	22.15 ± 1.69	Apr to May 2019	SAPS	IPAQ-SF	-0.335
NumanoğluAkbaş et al., 2020	Turkey	288 (129/159)	17 - 25	Jan to Jun 2019	SAS-SV	IPAQ-SF	-0.112
Buke et al., 2021	Turkey	300 (166/134)	21.36 ± 2.33	2020	SAS-SV	IPAQ	-0.271
Yang et al., 2021	China	608 (158/450)	20.06 ± 1.98	2020	MPATS	PARS-3	-0.109
Chen et al., 2022	China	9406 (3516/5890)	19.5 ± 1.07	Mar to Apr 2022	MPAI	IPAQ	-0.06
Guo et al., 2022	China	1433 (704/729)	19.67 ± 1.62	Dec 2020 to Feb 2021	MPATS	PARS-3	-0.158
Huang et al., 2022	China	452	23.65 ± 4.13	Aug to Sep 2021	BSMAS	IPAQ-SF	0.02

	China	452	23.65 ± 4.13	Nov to Dec 2021	BSMAS	IPAQ-SF	0.187
	China	452	23.65 ± 4.13	Feb 2021 to Apr 2022	BSMAS	IPAQ-SF	0.198
Saffari et al., 2022	China	391	22.85 ± 4.03	Aug to Sep 2021	SABAS	IPAQ-SF	-0.81
Tong et al., 2022	China	3609 (1891/1718)	/	September 2022	MPATS	IPAQ-SF	-0.173
Zeng et al., 2022	China	1843	19.75 ± 1.3	Oct to Nov 2021	MPATS	PARS-3	-0.14
Zhao et al., 2022	China	257 (73/185)	18-28	the COVID-19	MPA-11	PARS-3	-0.995
Gong et al., 2023	China	643 (363/280)	19.68 ± 1.40	/	SAS	PARS-3	-0.3
Han et al., 2023	China	4959 (1878/3081)	/	September 2022	MPATS	PARS-3	-0.279
Jin et al., 2024	China	930 (272/658)	/	March to April 2023	MPDIS	PARS-3	-0.133
Liu et al., 2023	China	488 (370/118)	19.21 ± 1.22	/	MPATS	PARS-3	-0.21
Tong et al., 2023	China	4399 (2481/1918)	19.20 ± 2.98	Mar to May 2022	MPATS	PARS-3	-0.713
Zhu et al., 2023	China	823 (499/324)	18.55 ± 0.83	December 2022	SAS-SV	IPAQ-SF	-0.151

Ke et al., 2024	China	608 (288/320)	20.27 ± 1.69	/	MPATS	PARS-3	-0.124
Meng et al., 2024	China	3599 (1958/1641)	19.12 ± 1.05	Oct to Dec 2023	MPATS	PARS-3	-0.447
Song et al., 2024	China	2905 (1181/1724)	19.31 ± 0.95	April to May 2023	MPATS	PARS-3	-0.131
Tao et al., 2024	China	861 (453/408)	22.1 ± 4.3	Aug to Nov 2023	MAPI	PARS-3	-0.09
Wang et al., 2024 (a)	China	274 (146/128)	20.31	May 2022	SAS	IPAQ-SF	-0.224
	China	528	/	March 10–14, 2022	MPATS	PARS-3	-0.232
Wang et al., 2024 (b)	China	461	/	Sept 11–15, 2022	MPATS	PARS-3	-0.44
	China	414 (197/217)	20.60 ± 0.83	March 10–14, 2023	MPATS	PARS-3	-0.311
Wu et al., 2024	China	590 (272/318)	19.67 ± 1.48	March to May 2022	MPAI	IPAQ-SF	-0.21
Yin et al., 2024	China	2274 (743/1531)	19.18 ± 1.02	Oct 8 to Oct 30, 2023	SAS-SV	PARS-3	-0.367
Zhao et al., 2024	China	337 (170/167)	20.93 ± 1.68	/	SVA	PARS-3	-0.193
Zhu et al., 2024	China	4670 (1714/2956)	/	September 2022	MPATS	PARS-3	-0.101

Meng et al., 2025	China	4562 (3570/992)	19.59 ± 1.21	Mar to Jun 2024	MPATS	PARS-3	-0.732

Abbreviations: MPA, mobile phone addiction; PA, physical activity; SAPS, Smartphone Addiction Proneness Scale; IPAQ-SF, International Physical Activity Questionnaire–Short Form; IPAQ, International Physical Activity Questionnaires; SAS-SV, Short Form of the Smartphone Addiction Questionnaire; MPATS, Mobile Phone Addiction Tendency Scale; PARS-3**,** Physical Activity Rating Scale-3; MPAI, The Mobile Phone Addiction Scale; BSMAS, Bergen Social Media Addiction Scale; SABAS, Smartphone Application-Based Addiction Scale; MPA-11, scale compiled by Hong et al; SAS, Smart Phone Addiction Scale; MPDIS, The Chinese version of the Mobile Phone Dependence Index Scale; SVA, Short Video Addiction Scale for College Students.

Table S3. Details of the scoring criteria in the JBI appraisal checklist.

Study	JBI appraisal checklist items										Total score (%)	Overall risk of bias
	1	2	3	4	5	6	7	8	9	10		
Kim et al., 2015	2	1	1	1	2	2	2	2	2	1	16 (80)	Low
Haripriya et al., 2019	2	2	1	2	1	1	2	2	2	1	16 (80)	Low
NumanoğluAkbaş et al., 2020	2	1	1	2	1	1	2	2	2	1	15 (75)	Low
Buke et al., 2021	2	2	2	2	1	1	2	2	2	1	17 (85)	Low
Yang et al., 2021	2	2	2	2	2	1	2	2	2	1	18 (90)	Low
Chen et al., 2022	2	2	2	2	2	1	2	2	2	1	18 (90)	Low
Guo et al., 2022	2	2	1	2	2	1	2	2	2	1	17 (85)	Low
Huang et al., 2022-1	2	2	2	2	2	1	2	2	2	1	18 (90)	Low
Huang et al., 2022-2	2	2	2	2	2	1	2	2	2	1	18 (90)	Low

Huang et al., 2022-3	2	2	2	2	2	1	2	2	2	1	18 (90)	Low
Saffari et al., 2022	2	2	2	1	2	1	2	2	2	1	17 (85)	Low
Tong et al., 2022	2	2	1	2	2	2	2	2	2	1	18 (90)	Low
Zeng et al., 2022	2	1	2	1	2	1	2	2	2	1	16 (80)	Low
Zhao et al., 2022	2	1	1	2	2	1	2	1	2	1	15 (75)	Low
Gong et al., 2023	2	1	2	1	2	1	2	2	2	1	16 (80)	Low
Han et al., 2023	2	2	2	1	2	1	2	2	2	1	17 (85)	Low
Jin et al., 2024	2	2	1	1	2	1	2	2	2	1	16 (80)	Low
Liu et al., 2023	2	1	1	1	2	1	2	2	2	1	15 (75)	Low
Tong et al., 2023	2	1	2	2	2	1	2	2	2	1	17 (85)	Low
Zhu et al., 2023	2	2	1	2	2	1	0	2	2	1	15 (75)	Low
Ke et al., 2024	2	2	2	2	2	1	2	2	2	1	17 (85)	Low

Meng et al., 2024	2	1	1	2	2	1	2	2	2	1	16 (80)	Low
Song et al., 2024	2	2	1	2	2	1	2	2	2	1	17 (85)	Low
Tao et al., 2024	2	2	2	2	2	1	0	2	2	1	16 (80)	Low
Wang et al., 2024 (a)	2	2	2	2	2	0	2	2	2	1	17 (85)	Low
Wang et al., 2024 (b)-1	1	2	1	1	2	0	2	2	2	2	15 (75)	Low
Wang et al., 2024 (b)-2	1	2	1	1	2	0	2	2	2	2	15 (75)	Low
Wang et al., 2024 (b)-3	1	2	1	1	2	0	2	2	2	2	15 (75)	Low
Wu et al., 2024	2	2	1	1	2	1	1	2	2	1	15 (75)	Low
Yin et al., 2024	2	2	2	2	2	0	2	2	2	1	17 (85)	Low
Zhao et al., 2024	2	1	0	1	2	0	2	2	2	2	14 (70)	Low
Zhu et al., 2024	2	2	0	1	1	0	2	2	2	2	14 (70)	Low
Meng et al., 2025	2	2	1	2	2	1	2	2	2	2	18 (90)	Low

Abbreviations: JBI, Joanna Briggs Institute.

Figure S1. Funnel plot of MPA

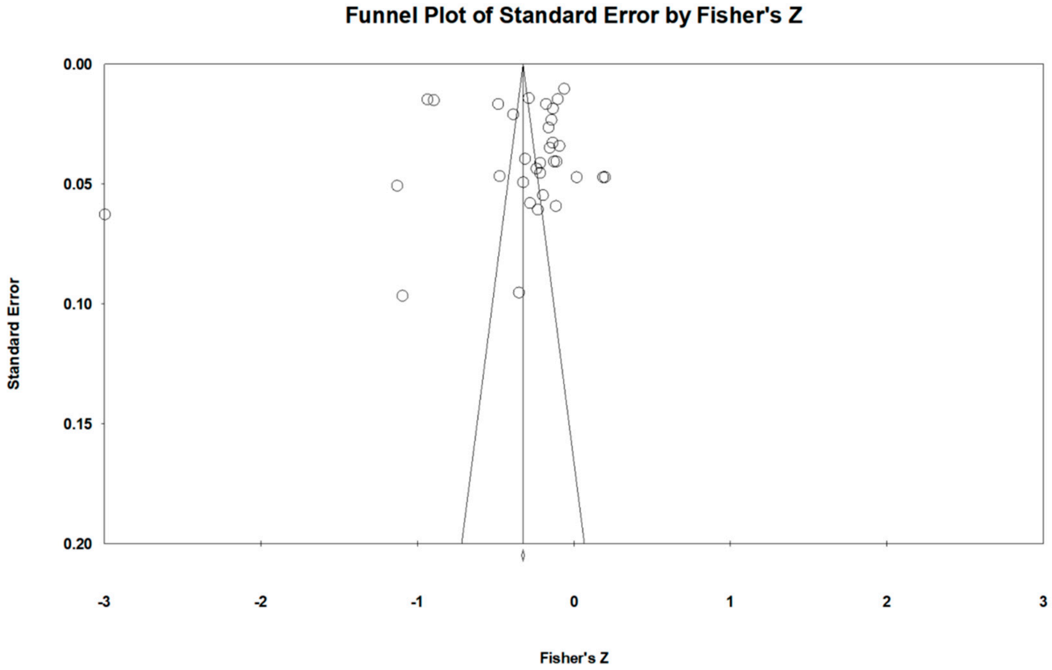

Figure S2. Sensitivity analysis results of MPA

**Studies**	**Z value**	***p* value**	**Correlation (95% CI) with study removed**
Kim et al., 2015	−5.155	<0.001	−0.330 (−0.440, −0.209)
Haripriya et al., 2019	−5.463	<0.001	−0.349 (−0.459, −0.230)
Numano luAkba et al., 2020	−5.564	<0.001	−0.356 (−0.464, −0.236)
Buke et al., 2021	−5.482	<0.001	−0.351 (−0.461, −0.231)
Yang et al., 2021	−5.553	<0.001	−0.356 (−0.465, −0.236)
Chen et al., 2022	−5.436	<0.001	−0.357 (−0.469, −0.235)
Guo et al., 2022	−5.487	<0.001	−0.355 (−0.465, −0.234)
Huang et al., 2022-1	−5.632	<0.001	−0.359 (−0.468, −0.240)
Huang et al., 2022-2	−5.738	<0.001	−0.364 (−0.471, −0.246)
Huang et al., 2022-3	−5.746	<0.001	−0.364 (−0.471, −0.246)
Saffari et al., 2022	−5.173	<0.001	−0.328 (−0.438, −0.208)
Tong et al., 2022	−5.377	<0.001	−0.354 (−0.466, −0.231)
Zeng et al., 2022	−5.482	<0.001	−0.355 (−0.465, −0.234)
Zhao et al., 2022	−4.903	<0.001	−0.275 (−0.376, −0.168)
Gong et al., 2023	−5.447	<0.001	−0.350 (−0.460, −0.230)
Han et al., 2023	−5.227	<0.001	−0.351 (−0.466, −0.225)
Jin et al., 2024	−5.525	<0.001	−0.355 (−0.465, −0.235)
Liu et al., 2023	−5.503	<0.001	−0.353 (−0.462, −0.233)
Tong et al., 2023	−5.621	<0.001	−0.334 (−0.437, −0.222)
Zhu et al., 2023	−5.519	<0.001	−0.355 (−0.464, −0.235)
Ke et al., 2024	−5.544	<0.001	−0.355 (−0.465, −0.236)
Meng et al., 2024	−5.240	<0.001	−0.346 (−0.459, −0.222)
Song et al., 2024	−5.446	<0.001	−0.355 (−0.466, −0.233)
Tao et al., 2024	−5.555	<0.001	−0.356 (−0.466, −0.237)
Wang et al., 2024	−5.507	<0.001	−0.353 (−0.462, −0.233)
Wang et al., 2024-1	−5.489	<0.001	−0.352 (−0.462, −0.232)
Wang et al., 2024-2	−5.385	<0.001	−0.346 (−0.456, −0.226)
Wang et al., 2024-3	−5.454	<0.001	−0.350 (−0.460, −0.230)
Wu et al., 2024	−5.497	<0.001	−0.353 (−0.462, −0.233)
Yin et al., 2024	−5.323	<0.001	−0.348 (−0.460, −0.226)
Zhao et al., 2023	−5.520	<0.001	−0.353 (−0.463, −0.234)
Zhu et al., 2024	−5.420	<0.001	−0.356 (−0.468, −0.233)
Meng et al., 2025	−5.744	<0.001	−0.332 (−0.433, −0.224)
**Overall**	−5.543	<0.001	−0.349 (−0.457, −0.231)

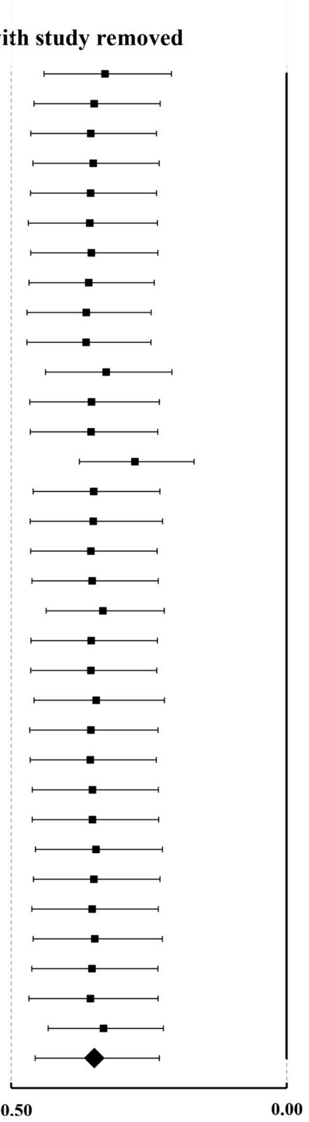

Supplement: Supplementary file 1 [file behavsci-15-01325-s001.zip › behavsci-3850502-supplementary.pdf]
